# Food Bioactive for Gut-Metabolic Axis Regulation and Microbiota Modulation

**DOI:** 10.3390/foods14152617

**Published:** 2025-07-26

**Authors:** Xiaoyan Liu, Tianjiao Wang, Ziwei Liu, Guangsen Fan

**Affiliations:** School of Food and Health, Beijing Technology and Business University, Beijing 100048, China; liuxiaoyan8112@163.com (X.L.); wangtianjiao1007@163.com (T.W.); 18333260154@163.com (Z.L.)

The escalating global burden of metabolic diseases, immune dysfunction, and age-related degeneration underscores the imperative for innovative nutritional interventions. Building upon foundational research in functional foods and flavor chemistry, this compilation, Dietary Fiber and Gut Microbiota, presents eight pioneering studies elucidating the mechanisms and therapeutic potential of diverse bioactive compounds, probiotics, prebiotics, and dietary fibers. Collectively, they highlight the gut ecosystem’s pivotal role as an interface between dietary components and systemic health.

A cornerstone study developed an innovative in vitro gut-liver-adipose axis model using a Transwell^®^ co-culture system (Caco-2/HepG2/3T3-L1). It demonstrated that combining specific probiotics (*Bifidobacterium bifidum* GM-25, B. infantis GM-21, *Lacticaseibacillus rhamnosus* GM-28) with polycosanols synergistically enhanced intestinal barrier integrity (increased TEER, upregulated tight junction proteins), reduced hepatic lipid accumulation (modulating CD36, SREBP-1, PPARγ, AMPK), and activated thermogenic pathways in adipocytes (elevating UCP1/PGC-1α). This underscores the promise of multi-targeted approaches for metabolic syndrome, aligning with broader efforts to identify bioactive combinations [1,2]. Complementing this, research on prebiotic dietary fibers revealed how gut microbial consortia metabolize polysaccharides from grains and mushrooms into distinct short-chain fatty acid (SCFA) profiles (acetate, propionate, butyrate). Metagenomic analysis linked taxonomic and enzymatic diversity to SCFA ratios, which directly modulated mucin secretion and epithelial cell differentiation in HT-29 cells, emphasizing the direct microbial influence on gut barrier function—a critical factor in metabolic health [3,4].

The profound impact of food processing on bioactivity is exemplified by work on yak milk proteins. Different heat treatments (low/high-temperature pasteurization, sterilization) induced varying degrees of protein oxidation, significantly altering murine gut microbiota composition (66 genera changed) and fecal metabolomes. Metagenomic and metabolomic analyses revealed that moderate oxidation upregulated pathways related to amino acid metabolism and energy homeostasis, suggesting context-dependent bioactivity influenced by processing [5]. Similarly, low molecular weight polysaccharides from *Laminaria japonica* (LJOO) exhibited significant hypoglycemic effects in type 2 diabetic mice. LJOO reduced fasting blood glucose and insulin, improved gut microbiota dysbiosis (increased Bacteroidetes/Firmicutes ratio, enriched SCFA producers like Lactobacillus, Bifidobacterium), and elevated cecal SCFA levels, demonstrating the gut-liver axis as a key mediator for anti-diabetic effects [6].

The potential of microbial-polysaccharide synergies for anti-aging was explored. A complex of *Agrocybe aegerita* polysaccharides (AAPS) and *Bifidobacterium lactis* Bb-12 extended the lifespan of *Drosophila melanogaster*, enhanced antioxidant capacity, and restored gut microbial structure in D-galactose-induced aging mice, notably increasing beneficial Lactobacillus abundance. This highlights the role of gut microbiota modulation in mitigating oxidative stress and age-related decline [7]. Furthermore, a formulation of *Ganoderma lucidum*, *Grifola frondosa*, and American ginseng (JGGA) enhanced immunity in cyclophosphamide-immunosuppressed mice. JGGA modulated gut microbiota (decreased Firmicutes/Proteobacteria, increased Bacteroidetes) and altered 30 fecal metabolites, linking microbial shifts and metabolic reprogramming to improved immune markers (thymic/splenic indices, cytokine levels), providing a mechanism for its immunomodulatory effects [8].

Dietary fibers from rice bran meal (RBDF), extracted via three methods, demonstrated significant hypolipidemic activity. Aqueous enzymatic extraction yielded RBDF (E-RBDF) with superior physicochemical properties (water/oil retention, cholesterol/bile salt adsorption). In vivo, all RBDFs reduced serum triglycerides, total cholesterol, LDL-C, and liver steatosis in hyperlipidemic rats while elevating HDL-C and hepatic antioxidant enzymes (SOD, GSH-Px), confirming their role in lipid management and oxidative stress reduction [9]. Beyond metabolic health, probiotic supplementation was reviewed for mitigating exercise-induced multi-organ stress via the gut-brain and gut-muscle axes. Probiotics improve barrier function, energy metabolism, redox balance, and neuroendocrine signaling, positioning them as viable ergogenic aids by modulating systemic stress responses [10].

Despite these significant advances, critical challenges mirror broader limitations in functional food research. Clinical validation remains sparse, sensory attributes and consumer acceptance are largely unaddressed, and causal links between specific molecules, microbiota shifts, and health outcomes demand deeper mechanistic exploration. Future research must prioritize clinical trials linking biomarkers to tangible health outcomes, integration of sensory science to ensure palatability alongside efficacy, and leveraging emerging technologies like AI for predictive bioactivity modeling and nanotechnology for targeted delivery.

This Special Issue illuminates the dynamic frontier of utilizing food-derived components—probiotics, prebiotics, polysaccharides, and functional fibers—to target the intricate gut-metabolic axis and microbiota. The findings reinforce diet as a master regulator of host physiology and provide a robust scientific foundation for developing next-generation functional foods against obesity, diabetes, aging, and immune dysregulation.

## References

[1] Chandrakar N., Kaur J., Banerjee M. (2024). Synergies of Bioactivities, Mechanisms, Dietary Factors and Functional Food Applications of Medicinal Insulin Plant (*Costus pictus D.*): A Review. Int. J. Food Sci. Technol..

[2] Wang W., Liu Y., Liu D., Zhou H., Li Y., Yuan W., Xu S., Wang J., Liang X., Weng J. (2024). Profiling of Antidiabetic Bioactive Flavonoid Compounds from an Edible Plant Kudzu (*Pueraria lobata*). J. Agric. Food Chem..

[3] Kim H., Kim B., Holzapfel W., Kang H. (2023). *Lactiplantibacillus plantarum* APsulloc331261 (GTB1™) Promotes Butyrate Production to Suppress Mucin Hypersecretion in a Murine Allergic Airway Inflammation Model. Front. Microbiol..

[4] Becht J., Kohlleppel H., Schins R., Kmpfer A. (2024). Effect of Butyrate on Food-Grade Titanium Dioxide Toxicity in Different Intestinal In Vitro Models. Chem. Res. Toxicol..

[5] Fu Y., Zhang Y., Soladoye O., Aluko R. (2020). Maillard Reaction Products Derived from Food Protein-derived Peptides: Insights into Flavor and Bioactivity. Crit. Rev. Food Sci..

[6] Ye J., Wu Z., Zhao Y., Zhang S., Liu W., Su Y. (2022). Role of Gut Microbiota in the Pathogenesis and Treatment of Diabetes Mullites: Advanced Research-Based Review. Front. Microbiol..

[7] Ling Z., Liu X., Cheng Y., Yan X., Wu S. (2022). Gut Microbiota and Aging. Crit. Rev. Food Sci..

[8] Petit J., Bruijn I., Goldman M., Erik V., Pellikaan W., Forlenza M., Wiegertjes G. (2022). β-Glucan-Induced Immuno-Modulation: A Role for the Intestinal Microbiota and Short-Chain Fatty Acids in Common Carp. Front. Immunol..

[9] Pradeep S., Srinivasan K. (2017). Amelioration of Oxidative Stress by Dietary Fenugreek (*Trigonella foenum-graecum* L.) Seeds is Potentiated by Onion (*Allium cepa* L.) in Streptozotocin-Induced Diabetic Rats. Appl. Physiol. Nutr. Metab..

[10] Liu Y., Hu Y., Ma B., Wang Z., Wei B. (2025). Gut Microbiota and Exercise: Probiotics to Modify the Composition and Roles of the Gut Microbiota in the Context of 3P Medicine. Microb. Ecol..

